# An Audit of Inpatient Consultation-Liaison Psychiatry Services at an Inner New York City Safety Net Hospital

**DOI:** 10.7759/cureus.34801

**Published:** 2023-02-09

**Authors:** Tarika Nagi, Saurabh Somvanshi, Gautam Shanmuga Dharmar Balasubramania Pandian, Subbulakshmi Mohan, Brian Altonen

**Affiliations:** 1 Psychiatry, Columbia University College of Physicians and Surgeons, Harlem Hospital Center, New York, USA; 2 Psychiatry and Behavioral Sciences, Columbia University College of Physicians and Surgeons, Harlem Hospital Center, New York, USA; 3 Data Analysis and Statistics, New York City Health and Hospital Corporation, New York, USA

**Keywords:** hospital-based medicine, consultation-liaison psychiatry, multidisciplinary teams, physician satisfaction, quality improvement research, inner city safety net hospital, mental health and psychiatry, process & performance improvement

## Abstract

Background

Our study’s primary objective is to audit the resource utilization of a consultation-liaison (CL) psychiatry service in an inner New York City safety net hospital. This cross-sectional, observational study was conducted as a subset of a quality improvement project at the hospital to investigate the characteristics of the emergent nature of consults, types, and the specialty from which the referral was placed to the CL services. This study aims to improve the efficacy of our consult process by improving the appropriateness and precision of consult requests.

Methodology

This cross-sectional, observational study was reviewed and approved by the Institutional Review Board under a quality improvement exemption. The study investigated the EPIC electronic medical record data for characteristics of consult referrals in the third quarter of 2019 from July 1, 2019, to September 30, 2019. A total of 629 consults were recorded during this period. We excluded follow-up calls, duplicate data rows, and patients with missing data points; the final consults were 421. Patients who required more than one new consult (follow-up excluded) within 90 days were considered; thus, the total number of patients who were included in the study was 327.

Results

Of the 421 consults identified in the dataset for review, only 45.8% were valid consults, 32.8% were not valid, and 21.4% were uncertain. Further, the most common department from which consults were placed was Medicine (73.2%), followed by Surgery (12.8%), Obstetrics/Gynecology (9%), Critical Care (3.6%), and, finally, Pediatrics (1.4%).

Conclusions

The study overviews the quality of general consults for the CL psychiatry service and how the CL staff manages it. It also provides an idea about the number of consults that can be comprehensively addressed.

## Introduction

According to the Principles for Best Practice in Clinical Audit, a clinical audit is defined as “a quality improvement process that seeks to improve patient care and outcomes through a systematic review of care against explicit criteria and the implementation of change.” Extensive research has shown a higher prevalence of psychiatric and behavioral issues in hospitalized patients [1]. Addressing these issues is particularly important and is done by the Consultation-Liaison (CL) service [2]. CL psychiatry is unique among subspecialties of medicine as it exists at the interface of psychiatry, general medicine, surgery, neurology, obstetrics/gynecology (Ob/Gyn), and pediatrics [3,4]. CL provides psychiatry consultation for hospital inpatient services such as Medicine, Surgery, Ob/Gyn, and Pediatrics [2]. Clearly, there is a need to streamline the process of consults. However, due to a lack of consensus guidelines, the implementation of change is challenging [5].

The primary aim of our project is to conduct an audit and investigate the category, type, and specialty from which the referral was placed, thereby improving the efficacy of our consult process by improving the appropriateness and precision of consult requests. In addition, we investigate the most common psychiatric diagnosis being referred to, anticipating and, eventually, focusing resources on patients who need it the most.

## Materials and methods

This project was submitted to the NYC Health + Hospitals Institutional Review Board (IRB). The quality improvement (QI) project was approved as IRB exempt by the Director, Research Administration, NYC Health + Hospitals on 11/13/2019. This cross-sectional observational study was conducted as a subset of a QI project at the hospital to investigate the characteristics of the emergent nature of consults, types, and the specialty from which the referral was placed to the CL services. Follow-up consults and emergency consults were excluded from the study.

Harlem Hospital Center is one of the teaching hospitals under NYC Health + Hospitals [6]. It is a 272-bed acute care facility and a level 1 trauma center [5]. It is affiliated with Columbia University and has six residency programs [6,7]. Harlem Hospital has multiple specialty services, including a burn unit, an adult intensive care unit, a neonatal intensive care unit, a pediatric intensive care unit, and a cardiac care unit [6]. Harlem Hospital also provides ambulatory care services [6]. It is the largest hospital in central Harlem, serving a population of 146,309 [6,8]. Each year, on average, Harlem Hospital provides over 210,00 outpatient visits, 83,000 emergency visits, and 13,000 inpatient admissions [6].

**Figure 1:**
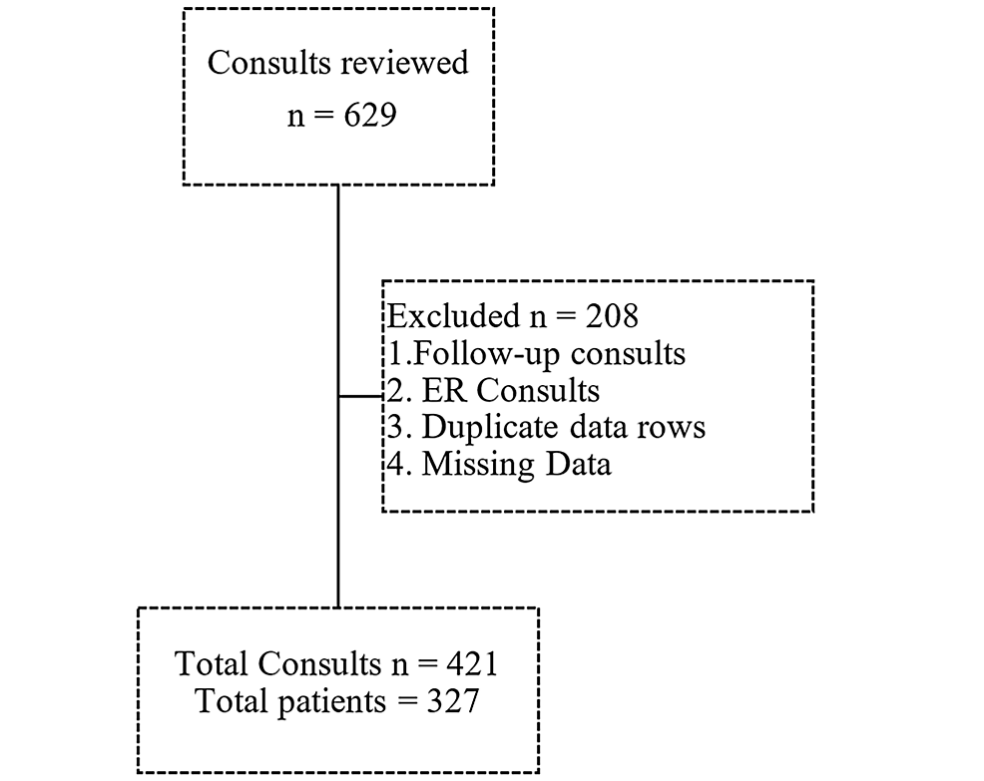
Flow diagram of exclusion criteria and the final number of consults.

The CL services address referrals from all inpatient departments in the hospital. All consults were evaluated by the CL team, including one attending and one PGY-2 resident. We investigated the referrals that were placed in the third quarter of 2019. The referral report was generated from EPIC (medical records system) for 90 days, from July 1, 2019, to September 30, 2019. A total of 629 consults were recorded during this period. The following data was collected from EPIC: the type of consult, the reason for the consult, if the resident team was contacted after placing the referral, the specialty requesting the consult, and patient details including past psychiatric history diagnosis, substance use, outpatient provider, discharge details, and forensic history. We also collected patient demographics, including age, gender, race, and domiciled history. The patient’s past psychiatric diagnosis was based on the Diagnostic and Statistical Manual of Mental Disorders (DSM-5) (American Psychiatric Association, 2013). We excluded follow-up calls, duplicate data rows, and patients with missing data points; the final consults were 421. Patients who required more than one new consult (follow-up excluded) within 90 days were considered; thus, the total number of patients who were included in the study was 327 (Figure 1).

Each variable taken into consideration for the study was further divided into categories and numbered in an Excel spreadsheet for analysis. The various categories for each variable are detailed in Table 1.

**Table 1:** Variables, categories, and coded response.

Variable	Categories	Coding
Type of consult	Routine	1
	Stat	2
Reason for consult	Valid	1
	Uncertain	2
	Not valid	3
Contacted resident team	Yes	1
	No	2
Gender	Male	1
	Female	2
	Other	3
Race	Black	1
	Hispanic	2
	Caucasian	3
	Asian	4
	South Asian	5
	Others	6
Domiciled history	Domiciled	1
	Domiciled other	2
	Homeless shelter	3
	Homeless street	4
Past psychiatric history	Depressive disorders	1
	Post-traumatic stress disorder and acute stress disorder	2
	Disruptive impulse control and conduct disorder	3
	Neurodevelopment disorders	4
	Alcohol-related disorders	5
	Anxiety disorders	6
	Schizophrenia spectrum and other psychotic disorders	7
	Somatic symptoms and related disorders	8
	Substance-related disorders other than alcohol	9
	Bipolar and related disorders	10
	Feeding and eating disorders	11
	Adjustment disorders	12
	Neurocognitive disorders	13
	Personality disorders	14
	No psychiatry history	15
	Others	16
Substance use	Yes	1
	No	2
Outpatient provider in psychiatry	Harlem outpatient services	1
	Outside services	2
	No follow-up	3
Disposition	Inpatient admission (Harlem Hospital)	1
	Discharge with Harlem outpatient appointment	2
	Discharge with no follow-up	3
	Discharge and follow-up with an outside provider	4
	Transfer to another inpatient facility	5
	Others	6
	Ongoing Consultation-Liaison follow-up	7
Specialty requesting consult	Medicine	1
	Surgery	2
	Pediatrics	3
	Obstetrics and Gynecology	4
	Critical Care	5
Forensic status	Forensic (Arrests/Handcuffs/AOT/State Hospital)	1
	No	2

Two datasets were developed (a patient dataset consisting of one row per patient/patient identifier, and a case dataset with one row of data per case). Data points were primarily unchanged for each patient, including age, gender, race, presence or absence of substance use disorder, specialty requesting consults, and patient domiciled or not. Infrequent changes and differences were noted for consult type, contacted team, disposition, specialty, and the presence or absence of forensic psychiatry considerations at the time of consult. The most likely feature to change was the consult type (emergent or not) and consult category, which was determined as part of this audit. The most changed portion of this data was a recording of the patient history data into binomial data (0 = No, 1 = Yes) to indicate whether the patient had the condition included in the list of numeric identifiers used for each chronic disease class. These two datasets were primarily evaluated using SPSS Statistics version 26 (IBM Corp., Armonk, NY, USA) applying standard descriptive chi-squared, analysis of variance, and linear or logistic regression applications. Some early analyses were performed using SAS version 9.4 (SAS Institute Inc, Cary, NC, USA).

​​​​​

## Results

A total number of 421 consults were identified over three months. Of the 421 consults identified in the dataset for review, only 45.8% were valid consults, 32.8% were not valid, and 21.4% were uncertain. The validity criterion was determined per the clinical judgment of the CL team. The consults were further divided into routine and stat; 86.5% were routine, and 13.5% were stat consults. Male patients had a slightly higher consult number, with 50.6%, compared with female patients, with 48.7%. African American was the most common race that was placed for consults, with a percentage of 64.1%, and most of the patients were domiciled, with a percentage of 75.1%.

**Figure 2:**
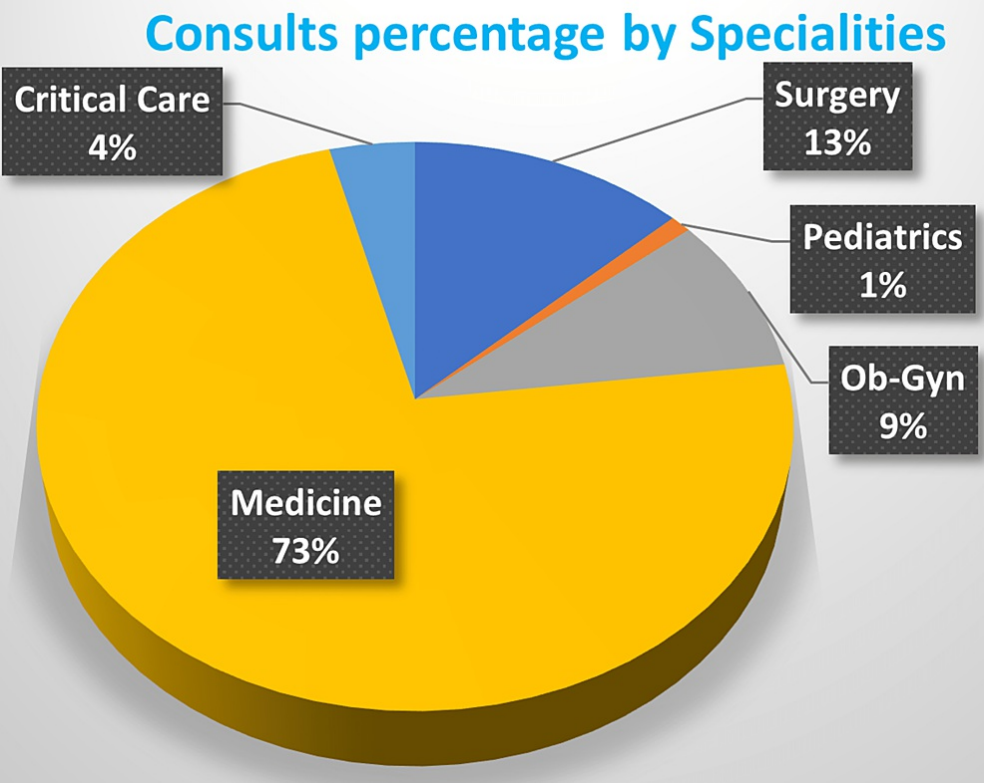
Consults by specialty.

The departments from which consults were placed in order of frequency were Medicine, with a percentage of 73.2%, followed by Surgery (12.8%), Ob/Gyn (9%), Critical Care (3.6%), and Pediatrics (1.4%) (Figure 2). Most of the time, the consulting team (91.7%) did not contact the resident team. Overall, 39.7% of the consults had some form of substance use in their medical history.

Assessed as per the Centers for Medicare and Medicaid Services (CMS) goals, post-discharge follow-up psychiatry appointment metrics were remarkable for the majority, with 61.8% of patients having/completing no follow-up appointments despite 55.6% of the consults requiring outpatient follow-up. The descriptive statistics are shown in Table 2, except for Age and Past psychiatric condition. There are a few missing data points in some variables labeled as Other in Table 2. Contacted resident team has one missing data, Gender has three missing data, and, finally, Outpatient psychiatry provider has one missing data.

**Table 2:** Descriptive statistics (n = 421).

Variables	Categories	Number	Percentage (%)
Type of consult	Routine	364	86.5
	Stat	57	13.5
Reason for consult	Valid	193	45.8
	Uncertain	90	21.4
	Not valid	138	32.8
Contacted resident team	Yes	33	7.8
	No	386	91.7
	Other	1	0.2
Gender	Male	213	50.6
	Female	205	78.7
	Other	3	0.7
Race	African American	270	64.1
	Hispanic	68	16.2
	Caucasian	37	8.8
	Asian	1	0.2
	South Asian	2	0.5
	Other	43	10.2
Domiciled history	Domiciled	316	75.1
	Domiciled other	46	10.9
	Homeless shelter	38	9.0
	Homeless street	21	5.0
Substance use	Yes	157	39.7
	No	254	60.3
Outpatient psychiatry provider	Harlem Hospital	33	7.8
	Outside services	127	30.2
	No follow-up	260	61.8
	Other	1	0.2
Disposition	Inpatient admission	25	5.9
	Discharge with OPD appointment	140	33.3
	Discharge follow-up not required	109	25.9
	Discharge follow-up with an outside provider	94	22.3
	Transfer to another inpatient facility	10	2.4
	Others	7	1.7
	Ongoing Consultation-Liaison follow-up	36	8.6
Specialty requesting consult	Medicine	308	73.2
	Surgery	54	12.8
	Pediatrics	6	1.4
	Obstetrics & Gynecology	38	9.0
	Critical Care	15	3.6
Forensic status	Yes	13	10
	No	408	90

**Figure 3:**
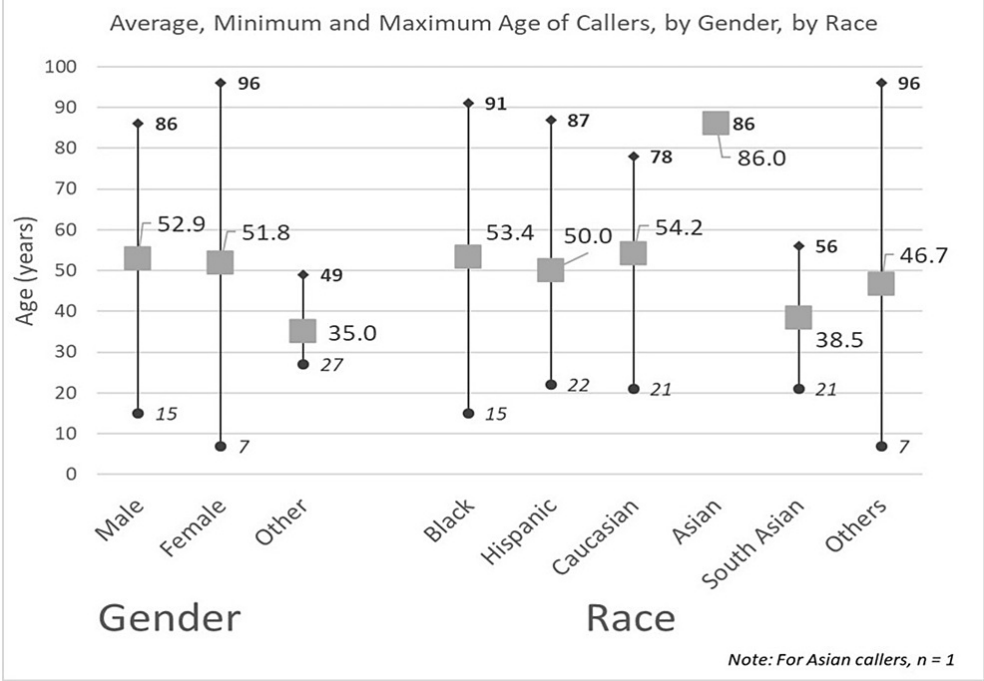
Age demographics against gender and race.

Age for all the 421 consults was calculated against Gender and Race, and the demographics of this are shown in Figure 3. The average age of all consults was 52.24 (range = 7-96, SD = 17.1). Age was divided into seven categories for the 327 patients (Table 3). Overall, 66.5% of the patients were above 45 years of age.

**Table 3:** Age of the patients (n = 327).

Age range (years)	Number (n)	Percentage (%)
18–25	27	8.2
26–35	47	14.3
36–45	35	10.7
46–55	71	21.7
56–65	77	23.5
65–75	42	12.8
75+	28	8.5

Past psychiatric condition was considered separately because most patients had two or more comorbid conditions, and the data were converted into binomial data (0 = No, 1 = Yes). The descriptive statistics for each past psychiatric condition are listed in Table 4.

**Table 4:** Past psychiatric condition.

Variable	Past psychiatric condition	Total number (n)	Percentage (%)
1	Depressive disorders	64	19.7
2	Post-traumatic stress disorder and acute stress disorders	22	6.8
3	Disruptive, impulse control, and conduct disorders	8	2.5
4	Neurodevelopmental disorders	4	1.2
5	Alcohol-related disorders	33	10.2
6	Anxiety disorders	24	7.4
7	Schizophrenia spectrum and other psychotic disorders	81	24.9
8	Somatic symptoms and related disorders	0	0
9	Substance-related disorders other than alcohol	40	12.3
10	Bipolar and related disorders	43	13.3
11	Feeding and eating disorders	2	0.6
12	Adjustment disorders	3	0.9
13	Neurocognitive disorder	4	1.2
14	Personality disorders	12	3.7
15	No psychiatry history	89	27.4
16	Other referral reasons and diagnoses	2	0.6

Summary statistics were developed for the natural language processing (NLP) data. These summary statistics focused on (a) the amount of data entered per patient visit, (b) the content of data entered per visit, in particular, related to the reason for the visit and the patient’s health status at the time, (c) patient behaviors related to visits with a focus on time between visits, and (d) underlying risks attached to these visits based upon text entries.

## Discussion

Type of consults

**Figure 4:**
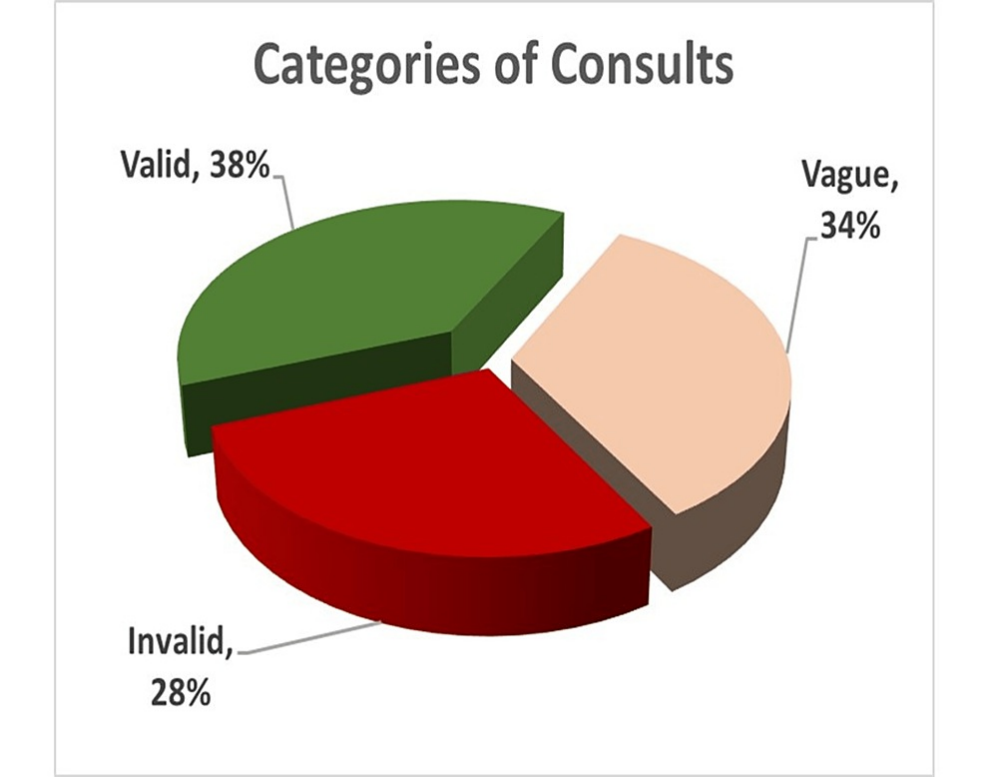
Pie chart showing consult categories.

The information entered for each visit is essential in that it documents the healthcare process and helps triage these patients and their care needs. In the best scenario, as a patient undergoes a visit, their healthcare needs are defined and met, thereby closing the case. Each visit has the potential of being inappropriate for the services provided by the psychiatrist, unmanageable by the psychiatrist, duplication of services already provided or while being managed, and appropriate for the psychiatrist and managed during the encounter. Consults were divided into three categories per the clinical judgment of psychiatry leadership by reviewing the text content of notes taken for each encounter. Examples for each type of consult are shown in Table 5, and a pie chart (Figure 4) shows the percentages of these consults.

**Table 5:** Examples of entries of case data.

Type of consult	Examples
Vague	Unspecified one to few word entries that may be interpreted too generically, i.e., “Capacity,” “Clearance,” “Reconciliation,” “Agitation,” “hx depression,” “Patient of Dr. N, talkative and requesting for his psychiatrist”
Invalid	Diagnosis given but without details, i.e., “schizophrenia,” “MAP,” “Mood/Anxiety/Psychiatric disorders,” “BPD,” “bipolar,” “substance abuse,” “depressed,” “f/u consult,” “change in mental status.” Those w/ assumed psych visit relationship: “Patient received a GSW to left forearm yesterday,” “Patient is newly diagnosed with HIV and was hospitalized three times for abdominal pain,” patient seen by psych. Please f/u.”
Valid	“Suicide” (one word-only entry, non-exclusion), “Please evaluate for depression,” “Self-destructive behavior”; those with no psych relationship may be for capacity for consent: “Pt has colon ca, please determine capacity for surgical consent,” “Pt s/p 20wk fetal demise,” “kindly evaluate 42-year-old for depression,” “flat affect, rule out postpartum depression,” “Patient with psychiatric history seen previously in CPEP,” “55-year-old male for jail clearance,” “postpartum patient with h/o anxiety. Please evaluate,” “psych meds reconciliation, prolonged QTC-484,” “Please evaluate this patient on antipsychotics complaining of throat closing up”

In theory, vague or invalid consults might occur due to the misunderstanding or misplaced expectations of the services requested of the CL team by the referring team or by the inadequate or partial documentation of a case by the referring team for various reasons. This requires evaluating the referral processes and, if possible, developing and implementing a method to place valid consults.

Repeat visits and changes in health

The numbers of people whose conditions appear to be escalating during their repeated visits were analyzed to look if there is an association. The individuals with repeat visits were categorized into the following four groups: (A) No change - individuals who did not change; (B) Mixed - individuals who demonstrated an increase in the complexity of their health condition by adding new clinical problems to their list; (C) Reducing - individuals who demonstrated a change toward reducing the severity or complexity of the case presentation; and (D) Escalating - individuals whose medical conditions resulted in a worsening or escalating of their health status.

Table 6 shows an evaluation of the repeat visitors in this dataset along with the counts for these groups. This evaluation method works with exceedingly small numbers for the dataset evaluated but still provides valuable insights. A patient’s problem history demonstrated a 16% (5/33 cases) escalation rate for those who underwent just two visits. The repeat visit cases with three or four visits total (n = 25) showed a worsening state of about 28-29%. This worsening or increase in risk is not linked to any specific conditions. This suggests that patients who visit more than two times should be treated as higher risk.

**Table 6:** Outcomes of visits focusing on escalating visit issues for repeat visitors.

Visits	Count	No change	Mixed	Reducing	Escalating	Percentage escalating
1	263	263	-	-	-	-
2	33	22	1	4	5	16%
3	18	5	7	1	5	28%
4	7	2	3	0	2	29%
5	2	0	2	0	0	0%
Total	323	293	11	5	12	21%

Time elapsed

The time elapsed between visits for multiple-visit patients was calculated and assessed [9]. Some patients had very brief lapses between their first two visits, such as less than a day. These time lapses ranged from 0.5 days to 45 days. The difference in dates for the two encounters was used to calculate the time-lapse. The emphasis of this review is on patients with two or more visits. From Table 7, it is clear that there is a trend that shows an increase in average time (days) between the visits.

**Table 7:** The period between visits for patients with more than one visit.

Visits	Patients	The average number of days between visits	Minimum number of days between visits	Maximum number of days between visits
1	263	-	-	-
2	33	4.8	1	33
3	18	9.4	1	43
4	7	11	3	22
5	2	14.5	10	19

Past psychiatric history

Most consults were placed for patients with no past psychiatric history (27.4%), followed by patients with a history of schizophrenia (24.9%) compared to the rest, as seen in Table 4. We also compared the past psychiatry history with the number of patient visits. We were able to find some interesting data which is shown in Table 8.

**Table 8:** Past psychiatric condition versus the number of patient visits.

Variable	Past psychiatric condition	One visit	Two visits	Three visits	Four visits	Five visits
1	Depressive disorders	54	5	3	1	0
2	Post-traumatic stress disorder and Acute stress disorders	18	1	2	1	0
3	Disruptive, impulse control, and conduct disorders	7	1	0	0	0
4	Neurodevelopmental disorders	3	0	1	0	0
5	Alcohol-related disorders	27	2	3	1	0
6	Anxiety disorders	22	2	1	0	0
7	Schizophrenia spectrum and other psychotic disorders	54	16	4	5	1
8	Somatic symptoms and related disorders	0	0	0	0	0
9	Substance-related disorders other than alcohol	33	4	1	1	0
10	Bipolar and related disorders	40	2	3	0	0
11	Feeding and eating disorders	2	0	0	0	0
12	Adjustment disorders	2	1	0	0	0
13	Neurocognitive disorder	2	0	1	0	0
14	Personality disorders	9	1	2	0	0
15	No psychiatry history	75	7	6	0	1
16	Other referral reasons and diagnoses	0	0	1	0	0

From Table 4 and Table 8, we can see that No psychiatry history has the most substantial number of consults, indicating most of the consults placed were Capacity assessment consults. Table 8 also shows patients suffering from Schizophrenia spectrum and other psychotic disorders had the most substantial number of repeat consults.

Validity and specialty requesting a consult

**Figure 5:**
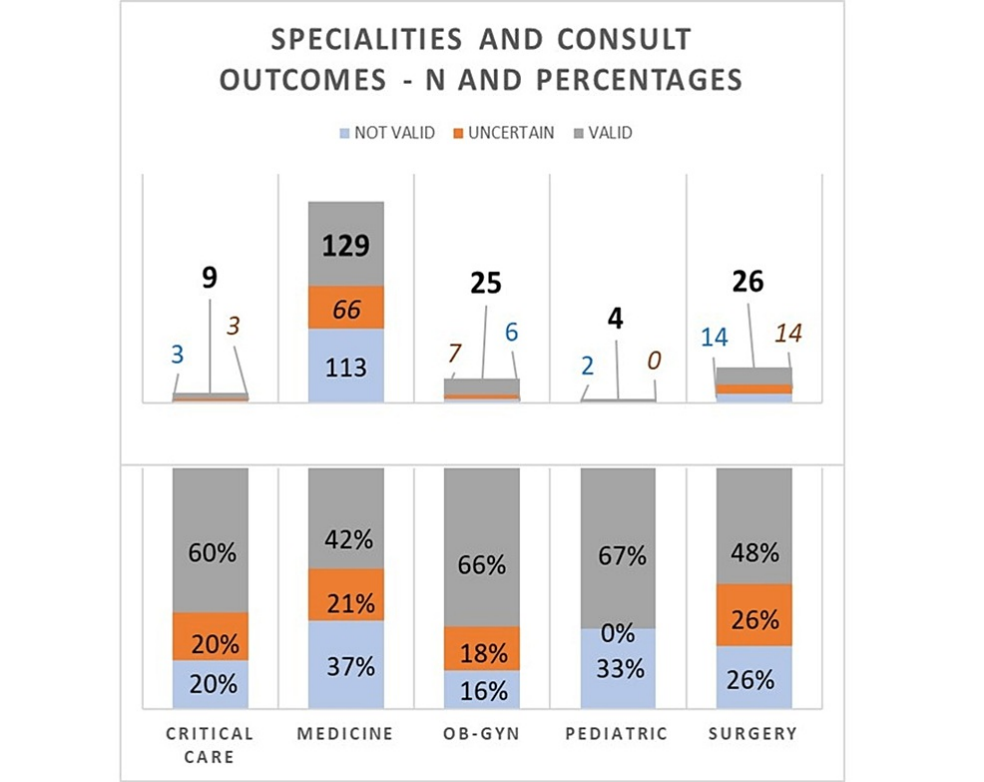
Specialties and consult outcomes.

The audit gave us insights into the specialty which placed the most consults. Most consults placed were from the Department of Medicine (308 consults, 74%), followed by Surgery (54 consults, 13%), Ob-Gyn (38 consults, 9%), Critical Care (15 consults, 4%), and, finally, Pediatrics (six consults, 1%). The validity of these consults was analyzed, as shown in Figure 5. We could observe that 37% (113 consults) placed by the Department of Medicine were not valid consults, followed by Pediatrics at 33%, Surgery at 26%, Critical Care at 20%, and Ob-Gyn at 16%. This data gives valuable insight into where to focus our attention.

Limitations

The audit was conducted only on one-quarter of the data. As only a segment of the year was evaluated, any seasonal differences in life experience and visiting behaviors may be missed. NLP processes, which are based on text content, are impacted by the data input behaviors of the consulting staff. Inter-rater reliability (the ability of two or more workers to evaluate the case with the same note-taking process) and standardized entry processes were not assessed for the involved staff. Because the data were only assessed for three months, certain variables had a small sample size data to have a meaningful impact. For example, two of the specialties in the dataset had a small number of rows of data, and the four and five-visit cases only had nine patients (n = 7 + 2 = 9 patients). Further, the study was conducted in a hospital-based, high-density, urban population setting which is not representative of suburban or non-community-based hospitals. Hence, these results need to be extrapolated on a larger scale with caution. There is no national benchmarking of CL service. Local services respond to local political and service demands [10].

Given the scale of psychiatric needs in general hospital inpatients, the case for providing inpatient CL psychiatry is not in doubt. CL psychiatry adds value to the general hospital in many ways: it provides education and support to medical and nursing staff, informs and shapes hospital policies and practices, and helps ward teams to provide care for individual patients [11]. Improving outcomes for patients with concurrent physical and mental health conditions is fundamental CL psychiatry work [12].

## Conclusions

The study gives an overview of the general consults that are placed to the CL department and how those consults were addressed by the CL service. It also gives an idea about the number of consults that can be comprehensively addressed. We receive a substantial number of consults not appropriate for an acute consult service, resulting in inequitable utilization of service resources. Time spent on inappropriate consults takes away from patients who genuinely need acute care. The collaborative council has sponsored psychiatry leadership to implement a multiphasic QI process. Phase 2 intervention of our study is planned as a pilot in the lead department, which will expand to other departments after refining the process based on pilot phase feedback.
